# Pilot study: impacts of cannabinoids from industrial hemp and repeated transportation events on cattle health and immune status

**DOI:** 10.1093/tas/txaf160

**Published:** 2025-12-08

**Authors:** Bailey R Fritz, Michael D Kleinhenz, Jason J Griffin, Mikaela M Weeder, Geraldine Magnin, Alyssa A Nelson, Blaine T Johnson, Andrew K Curtis, Johann F Coetzee

**Affiliations:** Department of Anatomy & Physiology, College of Veterinary Medicine, Kansas State University, Manhattan, KS, 66506, United States; Department of Clinical Sciences, College of Veterinary Medicine, Kansas State University, Manhattan, KS, 66506, United States; John C. Pair Horticulture Center, Kansas State University, Haysville, KS, 67060, United States; Department of Anatomy & Physiology, College of Veterinary Medicine, Kansas State University, Manhattan, KS, 66506, United States; Department of Anatomy & Physiology, College of Veterinary Medicine, Kansas State University, Manhattan, KS, 66506, United States; Department of Diagnostic Medicine/Pathobiology, College of Veterinary Medicine, Kansas State University, Manhattan, KS, 66506, United States; Department of Anatomy & Physiology, College of Veterinary Medicine, Kansas State University, Manhattan, KS, 66506, United States; Department of Diagnostic Medicine/Pathobiology, College of Veterinary Medicine, Kansas State University, Manhattan, KS, 66506, United States; Department of Anatomy & Physiology, College of Veterinary Medicine, Kansas State University, Manhattan, KS, 66506, United States; Department of Anatomy & Physiology, College of Veterinary Medicine, Kansas State University, Manhattan, KS, 66506, United States

**Keywords:** cannabinoids, cattle, industrial hemp, inflammation, stress, transportation

## Abstract

Recent legislative approval of industrial hemp (IH) cultivation has increased interest in the possibility of using IH and IH byproducts in livestock feed. Understanding the therapeutic effects of IH is critical for regulatory decisions and application to the cattle industry. The objective of this pilot study was to describe the effects of IH administration on stress and inflammatory biomarkers and activity in cattle experiencing repeated transportation. Twelve Holstein steers (430 kg, SEM = 3.4; 947 lb, SEM = 7.6) were assigned randomly to treatment sequences (*n* = 3 per sequence) in a 4 × 4 Latin Square design study (four periods and four treatment sequences). Treatments consisted of one of two drugs (IH or placebo; HEMP, PLBO) and one of two transportation events (transport or control; TRANS, CNTL) during each period so that every steer received all treatment combinations during the study. Industrial hemp was dosed at 5.5 mg/kg cannabidiolic acid (CBDA), with IH or placebo given once by oral bolus immediately prior to the transport or control event. Body weight, accelerometry, kinetic gait analysis, mechanical nociceptive threshold, infrared thermography, complete blood count (CBC), serum biochemistry, blood cortisol, prostaglandin E_2_ metabolite (PGEM), and serum amyloid A (SAA) were assessed using multiple linear regression. Summary statistics for plasma cannabinoid concentration were generated. There was a drug by transport by time interaction for change in PGEM from baseline (*P* = 0.03): steers in the HEMP-CNTL group had negative change at 48 h, whereas PLBO-CNTL steers had positive change at 48 h. Both TRANS and CNTL steers had increased lying times in the period immediately after transport. Steers in the TRANS group had greater body weight loss (*P* <0.0001), neutrophils (*P* *<* 0.0001), monocytes (*P* = 0.04), blood glucose (*P* < 0.0001), and total protein (*P* <0.0001) compared to CNTL steers immediately following transport. Cortisol area under the curve values were greater for TRANS versus CNTL steers (*P* < 0.0001). Lymphocyte concentrations were decreased for TRANS steers compared to CNTL following transport (*P* < 0.0001). There was a transport by time interaction for SAA (*P* < 0.0001), with TRANS steers at 24 and 32 h having the greatest SAA concentrations. Further investigation is required to confirm if feeding IH reduces PGEM. Novel outcomes evaluated in this pilot study will assist in design of future transportation trials.

## Graphical abstract

**Graphical Abstract:**
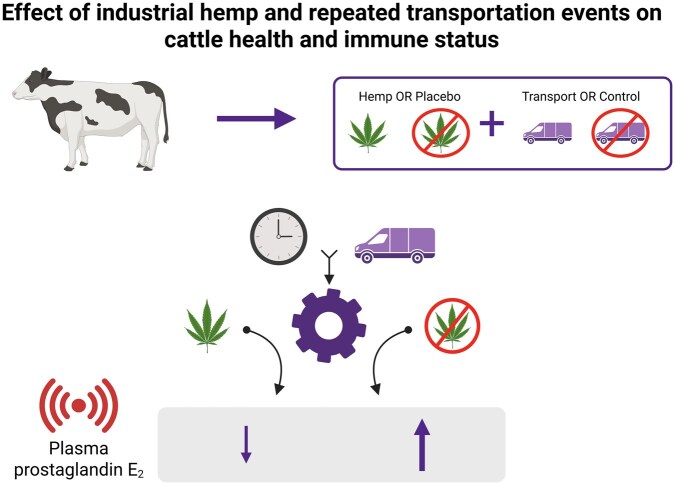

## Teaser

Administration of industrial hemp may reduce prostaglandin production in some cattle. Data from this study will enable future studies investigating IH in transported cattle to focus resources on relevant parameters.

## Introduction

Despite transportation being recognized as one of the most stressful events for livestock species, such as cattle (Vogel et al. 2019), most cattle in North America are transported at least once, and sometimes more than 5 times within their lives (Schwartzkopf-Genswein and Grandin 2019). A myriad of physiologic changes accompany the stress of transport and have been reviewed elsewhere (Van Engen and Coetzee 2018). Despite the welfare and economic implications of transport stress, Van Engen and Coetzee (2018) have reported that most interventions are applied after the transport event and generally consist of broad treatment of a group of animals with antibiotics (metaphylaxis) or vaccination. There is a clear need to establish effective means of reducing transport-related stress and subsequent disease in cattle.

Distinction of industrial hemp (IH)—*Cannabis sativa* containing ≤0.3% Δ9-tetrahydrocannabinol (9-THC), the psychoactive cannabinoid in marijuana—from marijuana has enabled legal cultivation of IH in the United States (Johnson 2021). Primary markets for IH and its fiber, seeds, oils, and cannabinoid extracts have garnered wide-reaching interest, from the construction sector to medicinal products (Kleinhenz et al. 2020b). Production of some IH byproducts, including hempseed products, oils, and cannabinoid extracts (eg cannabidiol, or CBD, oil), subsequently results in “waste” plant material. Cattle and other ruminants are efficient at digesting plant material and converting it into edible tissue products for consumers, such as milk and meat products. The nutrient profile, which is suitable for ruminants, along with the economic and environmental sustainability of using IH as a livestock feed has sparked interest from the livestock industry.

While previous work has investigated the plasma cannabinoid profiles of cattle administered a single dose of IH (Kleinhenz et al. 2020a) and stress and inflammatory changes in cattle administered a short-term course of IH (Kleinhenz et al. 2022), there is no published work evaluating the therapeutic potentials of IH in cattle. The pharmacodynamic properties of some cannabinoids, including roles in anti-inflammatory, analgesic, and antioxidant pathways (Takeda et al. 2008; Pellati et al. 2018; Rodríguez-Muñoz et al. 2018), make IH an attractive target for transport stress research.

The objective of this study was to describe the effects of repeated transport events and IH administration on stress and inflammatory biomarkers and activity levels in cattle.

## Materials and methods

### Ethics statement and animal disposition

Experimental procedures were approved by the Institutional Animal Care and Use Committee at Kansas State University (IACUC #4628). All study activities were conducted in conformity to requirements from the United States Department of Agriculture, the State of Kansas, and American Association for Accreditation of Laboratory Animal Care according to *The Guide for the Care and Use of Agricultural Animals in Research and Teaching* (ADSA ASAS PSA 2020). No enrolled steers were sold for human or animal consumption following trial termination.

### Animals and housing

Twelve (*n* = 12) Holstein steers, 1.5 years of age and weighing (± SEM) 430 ± 3.4 kg (947 ± 7.6 lb), were enrolled in December 2021. Steers had been previously acclimated to the research facility and were group-housed in outdoor pens with access to shelter throughout the study period. The pen area supplied per calf exceeded the guidelines established in the Guide for the Care and Use of Agricultural Animals in Research and Teaching. Steers were fed a custom grain mix twice daily (86.54% dry matter; 14.5% CP, 4.79% fat, 96.48 Mcal/cwt NE_m_, 65.91 Mcal/cwt NE_g_, 0.43% calcium, and 0.31% phosphorus on a dry matter basis) at 3% bodyweight per day at approximately 0900 and 1600 h and had ad libitum access to low-quality grass hay; diet met requirements for beef steers (NASEM 2016). The grain and hay fed to the steers is the standard diet used by the research facility for steers and bulls. Steers had ad libitum access to water via an automated watering device during the entire study.

### Experimental design

**Fig. 1.**
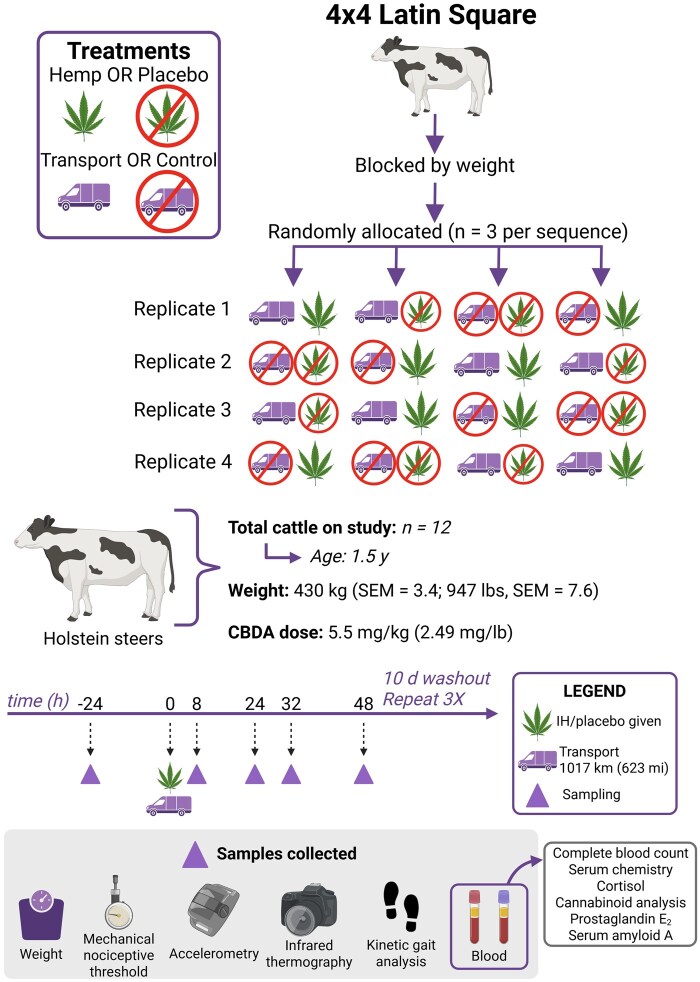
Diagrammatic representation of treatment sequences and study design. Created in BioRender. Fritz, B. (2025) https://BioRender.com/1ofwnvz and https://BioRender.com/9xrpvsi

Steers were blocked by body weight, and steers within blocks were assigned randomly to a treatment sequence using a random number generator (Excel; Microsoft Corp., Redmond, WA) in a 4x4 Latin Square design study (four periods and four treatment sequences) with 3 steers per treatment sequence. A 2x2 factorial treatment structure was used, with treatment combinations including (1) transport and industrial hemp (IH) administration, (2) transport and placebo administration, (3) control and IH administration, and (4) control and placebo administration [transport (TRANS), control (CNTL), IH administration (HEMP), placebo (PLBO)]. There were 4 periods of the study, with treatment sequence set so that no steer underwent transportation during consecutive periods of the study or more frequently than every 28 d. Steers underwent a 10-d washout period between periods to allow for elimination of cannabinoids. Due to the obvious nature of the treatments (transport and drug administration), investigators were not blinded; all investigators analyzing data were also present at sample collection. Baseline samples were collected at -24 h, with 0 h being the time of transport and IH or PLBO administration. Additional samples were collected at 8, 24, 32, and 48 h following transport. Steers in the TRANS groups were loaded onto a stock trailer immediately following IH or PLBO administration and were hauled for 623 mi (1017 km), or approximately 8 h. Steers in the CNTL groups were returned to their pen following IH or PLBO administration. Steers in the TRANS group did not have access to feed during transportation. A diagram of treatment sequences and study design is shown in Fig. 1.

### Industrial hemp dosing

Prior to study initiation, the cannabidiolic acid (CBDA) content as a percentage of total IH flower weight was determined using ultra-performance liquid chromatography triple quadrupole mass spectrometry (UPLC-MS/MS) and was used to calculate IH doses on an as-fed basis. Hemp flowers were submitted for extended cannabinoid panel analysis by the Kansas State University Olathe Campus Postharvest Physiology Lab (DEA registration no: RK0682256). Hemp content is presented in Table 1. Cattle in the HEMP groups received IH at a dose of 5.5 mg/kg CBDA by oral bolus. Hemp flower material was finely chopped using a kitchen-grade food chopper, weighed, and transferred into gelatin capsules. Steers were monitored after IH administration to ensure capsules were consumed (not spit out). Steers in the PLBO groups received an oral bolus with 10 to 15 g chopped alfalfa pellets contained in a gelatin capsule. Alfalfa pellets were chosen as they were the feed that was the most visually similar to IH and were readily accessible.

**Table 1.** Cannabinoid content (dry-matter basis) of the IH (cultivar: Endurance HT) administered in this 4-phase block randomized latin-square design study.^a^

	Mean	Standard deviation	Limit of detection
Cannabinoid	mg/g	mg/g	mg/g
**THC**	2.15	0.10	0.19
**THCA**	3.23	0.15	0.21
**CBD**	13.90	0.23	0.22
**CBDA**	79.88	2.64	0.28
**CBC**	1.85	0.13	0.09
**CBCA**	4.48	0.16	0.39
**CBGA**	2.06	0.03	0.52

a Holstein steers underwent repeat transport or control events and treatment with either a placebo or IH at a dose of 5.5 mg/kg CBDA given once by oral bolus immediately prior to the transport or control event.

Industrial hemp was grown and harvested by Kansas State University faculty at the John C. Pair Horticulture Center in Hays, Kansas. The cultivar Endurance HT was used (harvested October 2021). Industrial hemp was grown and handled in keeping with licensing requirements under the Kansas Department of Agriculture Industrial Hemp Research Program (license numbers: KDA-0621466839 and KDA-0302873296).

### Sample collection

Blood samples were collected 24 h prior to treatment administration and transportation and at 8, 24, 32, and 48 h after treatment administration. No samples were collected at 0 h; treatments were administered and transportation or control initiated at this timepoint. The 8 h timepoint was collected upon return of the TRANS groups. At each sampling timepoint, 17 mL of blood was collected via jugular or coccygeal venipuncture with an 18-gauge, 1.5-inch needle. Blood was collected into a 3 mL red-top tube (no additive), 2 mL EDTA tube, and two 6 mL heparinized tubes. Blood samples were stored on ice until transported back to the laboratory. The non-additive and EDTA tubes were submitted to the Kansas State Veterinary Diagnostic lab for serum biochemistry and complete blood count analysis according to American Association of Veterinary Laboratory Diagnosticians standards; non-additive tubes were allowed to clot. The heparinized tubes were centrifuged at 1,500 G for 10 min and each plasma sample was aliquoted into 4 1.5 mL cryovials and stored at -80°C until analysis for cortisol, PGEM, SAA, and cannabinoid concentrations.

### Body weight

Steers were weighed using an in-chute scale (TruTest, Datamars, Mineral Wells, TX, USA) 24 h prior to study initiation and at 0, 8, 24, 32, and 48 h during each study period. Change in body weight from 0 h was calculated for analysis. Feed intake was not monitored during this study.

### Accelerometry

IceQube accelerometer leg bands (IceRobotics, Edinburgh, Scotland, U.K.) were placed on the lateral aspect of the left hind limbs, just proximal to the metatarsophalangeal joint as recommended by the manufacturer directions. The accelerometers were placed one day before baseline (-24 h) data capture to allow steers to acclimate. Data capture was continuous throughout all treatment periods. Data were downloaded at the 48 h timepoint of each period, with steps, standing bouts, lying bouts, and motion index recorded, as described by Kleinhenz et al. (2022) and Martin et al. (2020). To account for expected differences in the transport period, parameters were summed across the following time periods: -24 to 0 h, 0 to 9 h, 9 to 24 h, and 24 to 48 h. All parameters were then converted to an hourly basis by dividing the sum by the corresponding number of hours for that period.

### Mechanical nociceptive threshold (MNT)

Mechanical nociceptive threshold (MNT; the minimum amount of force required to induce a withdrawal response) was collected on the lateral aspect of the coronary band on the lateral claw of both the left front and hind limbs, using methods described by Kleinhenz et al. (2017). Measurements were taken at 24 h prior to study initiation and at 8, 24, 32, and 48 h. A hand-held pressure algometer (Wagner Instruments, Greenwich, CT, USA) was used to apply force perpendicularly to the skin at a rate of approximately 1 kg/s. Force measurements were measured in triplicate for each limb at each timepoint and were recorded by a second investigator to eliminate measurement bias. If an output was the same for consecutive measurements, a new measurement was recorded until a unique force output was obtained. This was to reduce the chance of error with the algometer not recording a separate attempt. The mean of the three measurements was used for statistical analysis.

### Kinetic gait analysis

A commercially-available pressure and force measurement system (Strideway, Tekscan, Inc, South Boston, MA, USA) was used to analyze the gait of steers at -24 h, 8, 24, 32, and 48 h. Video synchronization was used to ensure correct identification of footfalls in the research software (Strideway 7.70, Tekscan, Inc., South Boston, MA, USA). Using methods described by Coetzee et al. (2014), stance time, stride length, force, force-time integral (FTI), and pressure were assessed in all limbs; for the forelimbs, gait distance and gait velocity were also assessed.

### Infrared thermography (IRT)

A thermography camera (TiX580 infrared camera, Fluke Corporation, Everett, WA, USA) was used to capture images of the medial canthus of the left eye and the left hind leg (at the level of the coronary band on the lateral claw) at -24, 8, 24, 32, and 48 h. Previous work has evaluated eye temperature as a non-invasive measure of stress (Lei et al. 2023). Coronary band temperature has been used as an indicator of lameness (Alsaaod and Büscher 2012; Alsaaod et al. 2014), and foot temperature is also impacted by physiologic and environmental stress and events such as feeding (Montanholi et al. 2008; Lei et al. 2023). The camera was calibrated prior to obtaining images and the ambient temperature and relative humidity were recorded from weather monitoring information for the area. The images were obtained by pointing the camera at the steer’s head or coronary band at a 45° angle and distance of 0.5 m. Images were analyzed for maximum temperature using research grade software (Fluke Smartview 4.3, Fluke Corporation, Everett, WA, USA) by drawing a 2 cm circle over the target area. For the hind limb images, the target area was drawn to include equal parts haired skin and hoof wall. The steers were restrained in a chute with a head catch for image acquisition.

### Plasma cortisol

Plasma cortisol concentrations were determined using a commercially available radioimmunoassay kit (MP Biomedicals, Santa Ana, CA, USA) following manufacturer specifications with minor modifications, as described previously (Martin et al. 2022). The standard curve was extended to include 1 and 3 ng/mL by diluting the 10 and 30 ng/mL manufacturer-supplied standards 1:10 respectively. The standard curve ranged from 1 to 300 ng/mL. A low (25 ng/mL) and high (150 ng/mL) quality control (QC) were run at the beginning and end of each set to determine inter-assay variability. Plain 12 × 75 mm polypropylene tubes were used as blank tubes to calculate non-specific binding. Input for standards, QC, and samples was adjusted to 50 µL. Samples were incubated at room temperature for 30 min prior to the addition of I-125. Manufacturer instructions were then followed. Tubes were counted on a Wizard2 gamma counter (PerkinElmer, Inc. Waltham, MA, USA) for 1 min. The raw data file was then uploaded onto MyAssays Desktop software (version 7.0.211.1238, MyAssays Ltd, Brighton, East Sussex, U.K.) for concentration determination. Standard curves were plotted as a 4-parameter logistic curve. Samples with a CV > 18% were re-analyzed, based on manufacturer recommendations. Inter- and intra-assay CV were 12.2% and 36.7%, respectively. The average LLOQ was 0.17 µg/dL. Average non-specific binding was 2.69%. Cortisol area under the curve (AUC) values for -24 to 24 h and -24 to 48 h were calculated using the trapezoidal method described by Lay et al. (1996); however, no baseline correction was included. Maximum concentrations (C_max_) for each individual steer per period were identified. The maximum and minimum observed C_max_ were recorded. Using Excel, the C_max_ values were log-transformed, and the mean, standard deviation, and 95% CI were calculated for each treatment group and across treatment groups. These summary statistics were then back transformed for reporting purposes.

### Plasma prostaglandin E_2_ metabolite (PGEM)

Prostaglandin E_2_ metabolites (PGEM) were analyzed using a commercially available ELISA kit (Cayman Chemical, Ann Arbor, MI, USA) following manufacturer specifications with minor modifications, as previously described (Martin et al. 2022). Sample input was adjusted to 375 µL with 1.5 mL ice-cold acetone added for sample purification. Samples were incubated at -20°C for 30 min, then centrifuged at 3,000 × g for 5 min. Supernatant was transferred to clean 13 × 100 mm glass tubes and evaporated using a CentriVap Concentrator (Labconco Corp., Kansas City, MO, USA) overnight (approximately 18 h). Samples were reconstituted with 375 µL of appropriate kit buffer. A 300 µL aliquot of the reconstituted sample was derivatized with proportionally adjusted kit components. Manufacturer protocol was then followed. Samples were diluted 1:2 and ran in duplicate. Absorbance was measured at 405 nm after 60 min of development (SpectraMax i3, Molecular Devices, LLC, San Jose, CA, USA).

Sample results were excluded if the raw read exceeded the raw read of the highest standard (Standard 1; 50 pg/mL) or was below the lowest acceptable standard. The lowest acceptable standard was defined for each individual plate and was identified by excluding standards that had a ratio of absorbance of that standard to the maximum binding of any well (%B/B_0_) of ≥80% or ≤20%. Any individual sample outside the standard curve, with a %B/B_0_ outside the 20–80% range, or a CV >15% were re-analyzed. If the CV exceeded 15% following re-analysis, the average of the CV values was recorded (samples were only re-analyzed twice). The inter-assay CV and intra-assay CV were 15.8% and 15.7%, respectively. The average LLOQ was 7.8 pg/mL. Data were analyzed using a commercially available data analysis tool (MyAssays Desktop).

### Serum amyloid a

Serum Amyloid A (SAA) concentrations were determined in plasma samples using a multispecies ELISA assay (Tridelta Development Ltd, Maynooth, County Kildare, IRE). Manufacturer specifications were followed, and samples were diluted as necessary. Absorbance was measured at 450 nm on a SpectraMax i3 plate reader. Raw data were analyzed using MyAssays Desktop software for concentration determination. Standard curves were plotted as a 4-parameter logistic curve. Samples with a CV > 15% were re-analyzed, based on manufacturer recommendations; if the CV exceeded 15% following re-analysis, the average of the CV values was recorded (samples were only re-analyzed twice). Inter-assay and intra-assay CV were 2.16% and 21.9%, respectively.

### Plasma cannabinoid concentrations

Plasma cannabinoids were measured as previously described (Kleinhenz et al. 2020a). Briefly, all solvents used such as methanol, acetonitrile, isopropanol, and formic acid were LCMS grade. Individual cannabinoid standards were purchased as solutions in methanol (Cerilliant Corp., Round Rock, TX, USA), including: (+)-11-nor-9-carboxy-Δ9-tetrahydrocannabinol glucuronide (THC-acid-glu), (−)-11-nor-9-carboxy-Δ9-tetrahydrocannabinol (THC-acid), (±)-11-hydroxy-Δ9-tetrahydrocannabinol (THC-11-OH), cannabidivarinic acid (CBDVA), cannabidivarin (CBDV), cannabidiol (CBD), cannabidiolic acid (CBDA), Δ9-tetrahydrocannabinolic acid A (THCA), cannabigerolic acid (CBGA), cannabigerol (CBG), Δ9-tetrahydrocannabinol (9-THC), Δ8-tetrahydrocannabinol (8-THC), cannabichromene (CBC), Δ9-tetrahydrocannabivarin (THCV), cannabinol (CBN). Cannabinoid analogs used as internal standards included ( ±)-cis-11-nor-9-carboxy-Δ9-tetrahydrocannabinol glucuronide-d_3_ (THC-glu-d_3_), cannabidiol-d_3_ (CBD-d_3_), Δ9-tetrahydrocannabinol-d_3_ (9-THC-d_3_), ( ±)-11-nor-9-carboxy-Δ9-tetrahydrocannabinol-d_9_ (THC-acid-d_9_), ( ±)-11-hydroxy-Δ9-tetrahydrocannabinol-d_3_ (THC-OH-d_3_), and cannabichromene-d_9_ (CBC-d_9_). All cannabinoids standards were kept in the freezer at − 20°C.

On the day of analysis, plasma samples were thawed at room temperature. Plasma, internal standard mixture (200 ng/mL), and acetonitrile with 0.1% formic acid were combined to precipitate plasma proteins. Internal standard was not added to the negative controls. Following vortexing and centrifugation, the supernatant was diluted with ultra-pure 18Ω water. Samples were then loaded onto a solid phase extraction plate using a nitrogen positive pressure manifold. Washes were performed with methanol: water (25:75) and eluted with acetonitrile: methanol (90:10). Eluates were diluted with water prior to analysis.

Cannabinoid analysis was performed using an Acquity H class UPLC and a TQ-S triple quadrupole mass spectrometer (Waters Corp., Milford, MA, USA). Chromatographic separation was achieved using an Eclipse Plus C18 UPLC column (100 × 2.1 mm, 1.8 µ, Agilent Technologies, Santa Clara, CA, USA) heated at 55°C. The mobile phase consisted of a gradient of water containing 0.1% formic acid (A) and acetonitrile (B) as follows: 0 min: 60% B, 6.50 min: 86% B, 7.50–9 min: 100% B, 9.01–12 min: 60% B. The flow rate was set at 0.5 mL/min, injection volume was 5 µL, and the run time per sample was 12 min. Data acquisition was performed using electrospray ionization in positive and negative mode using multiple reaction monitoring. Linear regression with a weighting factor of 1/X was used and accepted if the coefficient of correlation *R*^2^ was > 0.99. Calibration curves were linear from 0.1 to 100 ng/mL for all cannabinoids.

### Statistics

Data analysis was performed in JMP Pro (Version 16.0, SAS Institute Inc., Cary, NC, USA) using the distribution function for cannabinoid summary statistics and multiple linear regression for all other outcomes. Drug, transport, time, and their two-way and three-way interactions were treated as fixed effects. Block, period, and steer within period were treated as random effects. For IRT parameters, ambient temperature was also included as a random effect. Significance was set a priori at *P* *≤* 0.05. If the overall F-test for a response variable was significant, pairwise comparisons were performed using Tukey’s Honest Significant Difference adjustment for multiple comparisons, if there were more than 2 comparison groups. Figures were made using GraphPad (GraphPad Prism, 10.0, La Jolla, CA, USA).

## Results

**Fig. 2.**
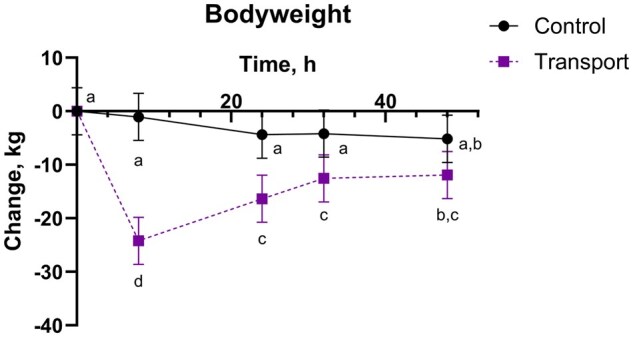
Body weight change (kg) (mean, 95% CI) of holstein steers undergoing repeat transport or control events and treatment with either a placebo or IH in a 4-phase block randomized latin-square design study. An alfalfa pellet placebo or IH, dosed at 5.5 mg/kg CBDA, was given once by oral bolus immediately prior to the transport or control event. Outcomes were assessed at -24, 0, 8, 24, 32, and 48 h relative to dosing and transport, with the 0 h body weight used as the baseline value for calculation of body weight change. Data points with different alphabetical identifiers are significantly different (*P* ≤ 0.05).

There was a transport by time interaction for body weight and body weight change (*P* *<* 0.0001), where TRANS steers at 8 h lost the most body weight (Fig. 2 and Table 2). Body weight for TRANS steers was different from baseline (both -24 and 0 h) subsequent timepoints through 48 h. On average, TRANS steers lost about 5% body weight (calculated using least squares means for TRANS at 8 h and the 0 h mean body weight).

**Table 2.** Body weight (kg), MNT (kg*force; kgf), and IRT (°C) outcomes (mean; 95% CI) of holstein steers undergoing repeat transport or control events and treatment with either a placebo or IH in a 4-phase block randomized latin-square design study.^a^

Parameter	Drug		Transport		*P*-value^b^						
Body weight	HEMP	PLBO	TRANS	CNTL	Dr	Tr	Ti	Dr*Tr	Dr*Ti	Tr*Ti	Dr*Tr*Ti
**Mean, kg**	439	439	435	443	0.97	0.51	<0.0001	0.93	0.31	<0.0001	1.00
	412 to 466	412 to 466	408 to 462	416 to 470							
**Change^c^, kg**	-6.9	-9.1	-13.0	-2.9	0.09	<0.0001	<0.0001	0.92	0.26	<0.0001	1.00
	-10.9 to -2.9	-13.1 to -5.0	-17.0 to -9.0	-7.0 to 1.1							
**MNT**											
**LF mean, kgf**	6.0	5.8	5.9	5.9	0.22	0.73	<0.0001	0.96	0.87	0.02	0.34
	2.5 to 9.4	2.3 to 9.3	2.4 to 9.3	2.4 to 9.4							
**LH mean, kgf**	5.0	4.9	4.9	5.1	0.54	0.08	<0.0001	0.70	0.16	0.08	0.48
	2.3 to 7.7	2.3 to 7.6	2.2 to 7.5	2.4 to 7.8							
**LF change^b^, %**	24.7	22.8	26.7	20.7	0.81	0.44	<0.0001	0.43	0.84	0.06	0.62
	-7.5 to 56.8	-9.4 to 54.9	-5.4 to 58.9	-11.4 to 52.8							
**LH change^b^, %**	24.9	28.3	25.5	27.6	0.64	0.77	<0.0001	0.51	0.40	0.17	0.72
	-30.7 to 80.5	-27.3 83.9	-30.1 to 81.1	-28.0 to 83.2							
**IRT**											
**L eye max, °C**	36.5	36.4	36.5	36.3	0.20	0.07	0.003	0.80	0.52	0.07	0.42
	35.9 to 37.1	35.7 to 37.0	35.9 to 37.2	35.7 to 37.0							
**L hoof max, °C**	20.3	19.9	20.0	20.2	0.53	0.80	0.009	0.71	0.13	0.47	0.23
	14.4 to 26.2	14.0 to 25.8	14.1 to 25.9	14.3 to 26.1							
**L eye change^b^, %**	1.2	0.9	1.8	0.4	0.49	0.001	0.0004	0.008	0.47	0.30	0.86
	-0.8 to 3.3	-1.1 to 3.0	-0.3 to 3.8	-1.7 to 2.4							
**L hoof change^b^, %**	40.7	38.6	37.4	41.9	0.76	0.51	<0.0001	0.34	0.20	0.93	0.69
	-79.0 to 160.3	-81.0 to 158.2	-82.3 to 157.0	-77.8 to 161.5							

a An alfalfa pellet placebo or IH, dosed at 5.5 mg/kg CBDA, was given once by oral bolus immediately prior to the transport or control event. Outcomes were assessed at -24, 8, 24, 32, and 48 h relative to dosing and transport. Body weight was additionally measured at 0 h.

b Drug—Dr; Transport—Tr; Time—Ti.

c ‘Change’ refers to change from baseline (0 h for body weight, -24 h for MNT and IRT) values for that parameter.

**Fig. 3.**
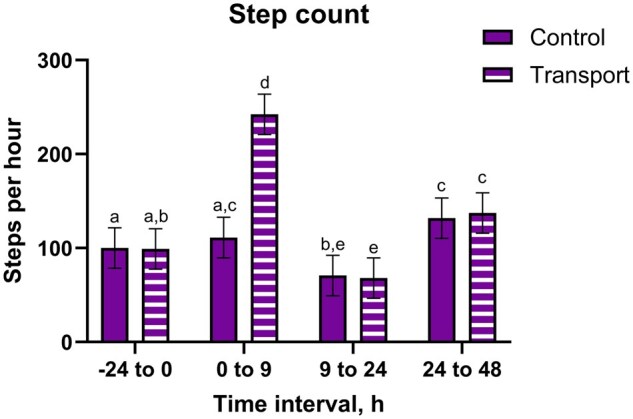
Step count data (mean, 95% CI) of holstein steers undergoing repeat transport or control events and treatment with either a placebo or IH in a 4-phase block randomized latin-square design study. An alfalfa pellet placebo or IH, dosed at 5.5 mg/kg CBDA, was given once by oral bolus immediately prior to the transport or control event. Accelerometric data were collected continuously throughout the study; data were collated into the following groups and converted into an hourly measurement: -24 to 0 h, 0 to 9 h, 9 to 24 h, and 24 to 48 h. Bars noted by different alphabetical identifiers are significantly different (*P* ≤ 0.05).

There were transport by time interactions (*P* *<* 0.0001) for motion index, step count, and standing ratio, where TRANS steers from 0 to 9 h having greater outcomes than other groups (Fig. 3 and Table 3). Lying ratio had a transport by time interaction (*P* *<* 0.0001), where TRANS steers from 0 to 9 h had the lowest lying ratios. Both TRANS and CNTL steers had greater lying ratios and lower step counts, standing ratios, and motion indices from 9 to 24 h compared to other periods (*P* *≤* 0.05). There was a transport by time interaction for lying bouts (*P* *<* 0.0001), where TRANS steers from 0 to 9 h and 9 to 24 had fewer lying bouts than CNTL steers from those intervals.

**Table 3.** Accelerometric parameters (mean; 95% CI) of holstein steers undergoing repeat transport or control events and treatment with either a placebo or IH in a 4-phase block randomized latin-square design study.^a^

Parameter	Drug		Transport		*P*-value^b^						
	HEMP	PLBO	TRANS	CNTL	Dr	Tr	Ti	Dr*Tr	Dr*Ti	Tr*Ti	Dr*Tr*Ti
**Motion index**	528	531	602	457	0.89	<0.0001	<0.0001	0.32	0.76	<0.0001	0.87
	426 to 630	429 to 634	500 to 704	355 to 560							
**Steps**	120	121	137	104	0.83	<0.0001	<0.0001	0.39	0.81	<0.0001	0.82
	99 to 140	100 to 141	116 to 157	83 to 124							
**Lying bouts**	0.38	0.37	0.30	0.46	0.54	<0.0001	<0.0001	0.79	0.51	<0.0001	0.77
	0.31 to 0.46	0.30 to 0.45	0.23 to 0.37	0.38 to 0.53							
**Standing ratio**	0.51	0.51	0.57	0.45	0.84	<0.0001	<0.0001	0.46	0.94	<0.0001	0.65
	0.49 to 0.53	0.49 to 0.53	0.55 to 0.59	0.42 to 0.47							
**Lying ratio**	0.49	0.49	0.43	0.55	0.96	<0.0001	<0.0001	0.39	0.94	<0.0001	0.71
	0.47 to 0.51	0.47 to 0.51	0.40 to 0.45	0.53 to 0.58							

a An alfalfa pellet placebo or IH, dosed at 5.5 mg/kg CBDA, was given once by oral bolus immediately prior to the transport or control event. Accelerometric data were collected continuously throughout the study; data were collated into the following groups and converted into an hourly measurement: -24 to 0 h, 0 to 9 h, 9 to 24 h, and 24 to 48 h.

b Drug—Dr; Transport—Tr; Time—Ti.

There was a transport by time interaction for left front average MNT value (*P* = 0.02), where CNTL and TRANS steers had lower baseline values than CNTL or TRANS steers at 32 or 48 h, CNTL steers at 8 h, or TRANS steers at 24 h (Table 2). There was a time main effect for left hind average MNT value (*P* *<* 0.0001), where values progressively increased from -24 to 48 h, with -24 and 8 h values being significantly lower than all other timepoints (*P* *≤* 0.05). The same temporal pattern was seen for the change in MNT values from baseline for both hind and front limbs (*P* *<* 0.0001), with greater positive percent change from baseline with increasing time.

There was a drug by transport interaction (*P* = 0.04) for the kinetic gait analysis parameter rear pressure (Table 4). However, post-hoc comparisons did not reveal any significant differences between groups (*P* *>* 0.05). All outcome parameters except front and rear stance and front pressure had a time main effect (*P* *≤* 0.009), with 8 h outcomes being the greatest and 48 h being the lowest, except for front gait distance, in which 32 h outcomes were the greatest.

**Table 4.** Kinetic gait analysis outcomes (mean; 95% CI) for holstein steers undergoing repeat transport or control events and treatment with either a placebo or IH in a 4-phase block randomized latin-square design study.^a^

Parameter	Drug		Transport		*P*-value^b^						
	HEMP	PLBO	TRANS	CNTL	Dr	Tr	Ti	Dr*Tr	Dr*Ti	Tr*Ti	Dr*Tr*Ti
**Front gait distance, cm**	149	147	146	150	0.81	0.37	0.006	0.53	0.95	0.92	0.24
	137 to 160	135 to 159	134 to 157	138 to 162							
**Front gait velocity, cm/s**	120	125	123	123	0.45	0.97	0.003	1.00	0.95	0.18	0.29
	107 to 133	113 to 138	110 to 136	110 to 136							
**Front stance, s**	0.88	0.83	0.87	0.84	0.35	0.49	0.16	0.84	0.67	0.47	0.49
	0.71 to 1.04	0.67 to 1.00	0.71 to 1.03	0.68 to 1.00							
**Front stride, cm**	145	148	147	145	0.51	0.58	<0.0001	0.81	0.95	0.93	0.90
	135 to 155	138 to 157	137 to 157	136 to 155							
**Front force, kg**	147	142	146	143	0.22	0.52	<0.0001	0.89	0.90	0.73	0.57
	143 to 151	137 to 146	141 to 150	139 to 148							
Front FTI, kg*s	87.3	80.7	84.6	83.4	0.11	0.77	<0.0001	0.94	0.92	0.39	0.26
	82.9 to 91.6	76.3 to 85.0	80.2 to 88.9	79.0 to 87.7							
**Front pressure, kg/cm^2^**	4.1	4.0	4.1	4.1	0.12	0.43	0.054	0.39	0.67	0.31	1.00
	3.6 to 4.6	3.6 to 4.5	3.6 to 4.6	3.6 to 4.6							
**Rear stance, s**	0.84	0.81	0.82	0.83	0.46	0.73	0.25	0.90	0.92	0.38	0.47
	0.78 to 0.90	0.75 to 0.87	0.76 to 0.88	0.77 to 0.89							
**Rear stride, cm**	146	145	147	145	0.75	0.54	<0.0001	0.74	0.90	1.00	0.43
	136 to 157	135 to 156	136 to 157	134 to 155							
**Rear force, kg**	132	133	130	135	0.97	0.26	<0.0001	0.68	0.76	0.30	0.66
	120 to 145	120 to 145	117 to 143	122 to 148							
Rear FTI, kg*s	76.7	72.7	74.3	75.2	0.42	0.85	0.0003	0.56	0.85	0.08	0.52
	65.0 to 88.4	61.1 to 84.4	62.6 to 85.9	63.5 to 86.9							
**Rear pressure, kg/cm^2^**	3.9	3.9	3.9	3.9	0.95	0.67	0.009	0.04	0.11	0.40	0.93
	3.4 to 4.4	3.4 to 4.5	3.4 to 4.4	3.4 to 4.5							

a An alfalfa pellet placebo or IH, dosed at 5.5 mg/kg CBDA, was given once by oral bolus immediately prior to the transport or control event. Outcomes were measured at -24, 8, 24, 32, and 48 h relative to dosing and transport.

b Drug—Dr; Transport—Tr; Time—Ti.

The left eye maximum temperature percent change from baseline had a drug by transport interaction (*P* = 0.008), where HEMP-TRANS steers had greater positive percent change than HEMP-CNTL and PLBO-CNTL groups (*P* *≤* 0.05) (Table 2). All IRT parameters had time main effects (*P* *≤* 0.009), but there was no pattern shared among the parameters regarding the hierarchy of least squares means.

**Fig. 4.**
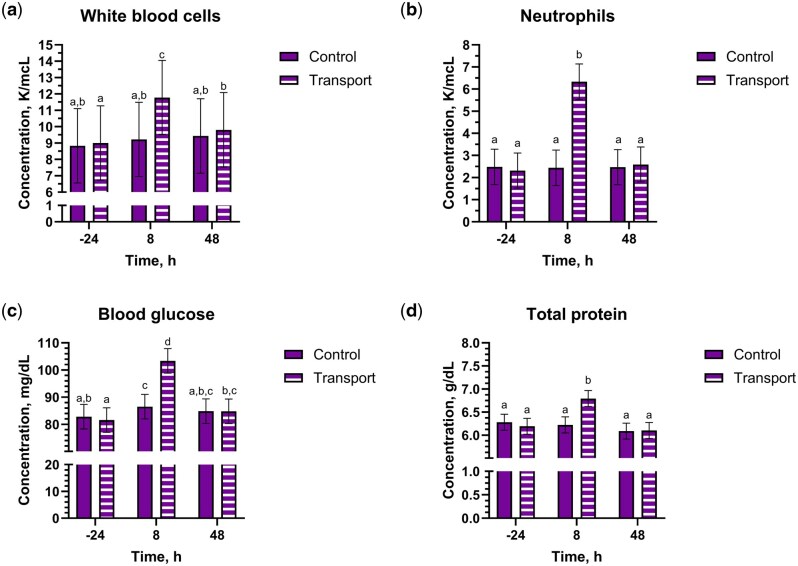
White blood cell count a), neutrophil count b), blood glucose c), and total protein d) outcomes (mean, 95% CI) for holstein steers undergoing repeat transport or control events and treatment with either a placebo or IH in a 4-phase block randomized latin-square design study. An alfalfa pellet placebo or IH, dosed at 5.5 mg/kg CBDA, was given once by oral bolus immediately prior to the transport or control event. Outcomes were measured at -24, 8, and 48 h relative to dosing and transport. Bars noted by different alphabetical identifiers are significantly different (*P* ≤ 0.05).

Blood glucose had a drug by transport by time interaction (*P* = 0.03), where PLBO-TRANS and HEMP-TRANS steers at 8 h had greater values than other groups and timepoints (*P* *≤* 0.05) (Fig. 4 and Table 5). A contrast between the PLBO-TRANS and HEMP-TRANS groups was not significant (*P* = 0.054). White blood cell, neutrophil, monocyte, and lymphocyte concentrations, hematocrit, and total protein all had transport by time interactions (*P* *≤* 0.04). White blood cell, neutrophil, and monocyte concentrations and total protein were greater for TRANS steers at 8 h; lymphocyte concentrations were the lowest for TRANS steers at 8 h. For hematocrit, CNTL steers at 8 h had significantly lower values than TRANS steers at 8 and 48 h or CNTL steers at -24 and 48 h (*P* *≤* 0.05).

**Table 5.** Select parameters (mean; 95% CI) from complete blood count and serum biochemistry analyses for holstein steers undergoing repeat transport or control events and treatment with either a placebo or IH in a 4-phase block randomized latin-square design study.^a^

		Drug		Transport		*P*-value^b^						
Parameter^c^	Reference	HEMP	PLBO	TRANS	CNTL	Dr	Tr	Ti	Dr*Tr	Dr*Ti	Tr*Ti	Dr*Tr*Ti
**WBC, K/µL**	4.9–12.0	9.8	9.6	10.2	9.2	0.61	0.01	<0.0001	0.84	0.42	<0.0001	0.81
		7.5 to 12.1	7.3 to 11.9	7.9 to 12.5	6.9 to 11.5							
**NEU, K/µL**	1.8–6.3	3.1	3.1	3.7	2.5	0.90	<0.0001	<0.0001	0.82	0.27	<0.0001	0.88
		2.3 to 3.9	2.3 to 3.9	3.0 to 4.5	1.7 to 3.2							
**MONO, K/µL**	0.0–0.8	0.6	0.6	0.6	0.6	0.93	0.75	0.01	0.86	0.96	0.04	0.53
		0.4 to 0.8	0.4 to 0.8	0.4 to 0.8	0.4 to 0.8							
**LYMPH, K/µL**	1.6–5.6	5.7	5.6	5.6	5.8	0.68	0.51	<0.0001	0.80	0.13	<0.0001	0.46
		3.9 to 7.6	3.7 to 7.5	3.7 to 7.4	3.9 to 7.7							
**Fibrinogen, mg/dL**	300–700	388	402	388	401	0.59	0.64	0.42	0.10	0.38	0.33	0.55
		323 to 452	338 to 466	324 to 453	337 to 465							
**HCT, %**	22–33	29	29	30	29	0.76	0.15	<0.0001	0.052	0.53	0.002	0.77
		28 to 31	28 to 31	28 to 31	28 to 31							
**Glucose, mg/dL**	29–73	87	88	90	85	0.26	<0.0001	<0.0001	0.30	0.68	<0.0001	0.03
		82 to 91	83 to 93	85 to 95	80 to 89							
**Total protein, g/dL**	6.0–9.0	6.3	6.3	6.4	6.2	0.84	0.03	<0.0001	0.32	0.90	<0.0001	0.13
		6.1 to 6.4	6.1 to 6.5	6.2 to 6.5	6.0 to 6.4							

a An alfalfa pellet placebo or IH, dosed at 5.5 mg/kg CBDA, was given once by oral bolus immediately prior to the transport or control event. Outcomes were measured at -24, 8, and 48 h relative to dosing and transport.

b Drug—Dr; Transport—Tr; Time—Ti.

c White blood cell concentration—WBC; Segmented neutrophil concentration—NEU; Monocyte concentration—MONO; Lymphocyte concentration—LYMPH; Hematocrit (calculated) – HCT.

**Fig. 5.**
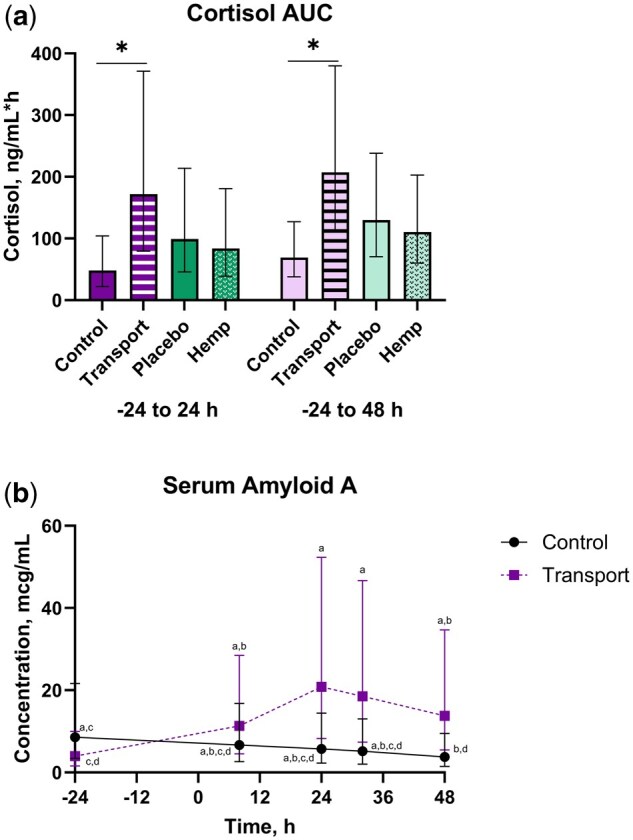
Cortisol AUC a) and SAA b) outcomes (mean, 95% CI) for holstein steers undergoing repeat transport or control events and treatment with either a placebo or IH in a 4-phase block randomized latin-square design study. An alfalfa pellet placebo or IH, dosed at 5.5 mg/kg CBDA, was given once by oral bolus immediately prior to the transport or control event. Cortisol and SAA were log-transformed for analysis and backtransformed for presentation. Data noted by different alphabetical identifiers or ‘*’ are significantly different (*P* ≤ 0.05).

There were transport and time main effects for average concentration (*P* *≤* 0.002), where TRANS steers had greater concentrations than CNTL (Table 6). Average cortisol was greater for 8 and 24 h compared to all other timepoints, with -24 and 48 h having the lowest concentrations. There were transport main effects for the AUC parameters (*P* *<* 0.0001), where TRANS steers had greater AUC values than CNTL (Fig. 5 and Table 6). The minimum and maximum observed cortisol C_max_ were 0.39 and 17.79 ng/mL, respectively. The mean C_max_ across groups was 3.87 ng/mL (95% CI: 2.92, 5.14 ng/mL). The mean C_max_ values (95% CI) for HEMP, PLBO, TRANS, and CNTL steers were 3.46 ng/mL (2.26, 5.30 ng/mL), 4.33 ng/mL (2.98, 6.31 ng/mL), 6.58 ng/mL (5.14, 8.44 ng/mL), and 2.28 ng/mL (1.51, 3.44 ng/mL), respectively.

**Table 6.** Cortisol, PGEM, and SAA outcomes (mean; 95% CI) for holstein steers undergoing repeat transport or control events and treatment with either a placebo or IH in a 4-phase block randomized latin-square design study.^a^

Parameter	Drug		Transport		*P*-value^b^						
Cortisol	HEMP	PLBO	TRANS	CNTL	Dr	Tr	Ti	Dr*Tr	Dr*Ti	Tr*Ti	Dr*Tr*Ti
**Average, ng/mL**	1.3	1.0	1.6	0.8	0.26	0.002	<0.0001	0.37	0.52	0.15	0.66
	0.8 to 2.1	0.6 to 1.7	0.9 to 2.6	0.5 to 1.4							
AUC (-24–24 h), ng/mL*h	84	99	172	48	0.51	<0.0001	N/A	0.81	N/A	N/A	N/A
	39 to 181	46 to 214	80 to 371	22 to 104							
AUC (-24–48 h), ng/mL*h	111	130	207	69	0.49	<0.0001	N/A	0.79	N/A	N/A	N/A
	60 to 203	71 to 238	113 to 380	38 to 127							
**PGEM**											
**Average, pg/mL**	18.7	16.8	17.3	18.1	0.17	0.55	0.02	0.76	0.17	0.62	0.11
	16.0 to 21.8	14.4 to 19.6	14.9 to 20.2	15.6 to 21.1							
**Change, %**	-4.5	5.3	-2.5	3.3	0.10	0.32	0.04	0.52	0.23	0.85	0.03
	-22.6 to 13.5	-12.7 to 23.3	-20.5 to 15.5	-14.8 to 21.3							
**SAA, µg/mL**	7.8	8.8	11.9	5.8	0.75	0.07	0.0001	0.34	0.89	<0.0001	0.20
	3.1 to 19.4	3.5 to 22.1	4.8 to 29.7	2.3 to 14.4							

a An alfalfa pellet placebo or IH, dosed at 5.5 mg/kg CBDA, was given once by oral bolus immediately prior to the transport or control event. Average cortisol, cortisol AUC -24 to 24 h, cortisol AUC -24 to 48 h, average PGEM, and average SAA were log-transformed for analysis. Means and 95% CI are presented as back-transformed values.

b Drug—Dr; Transport—Tr; Time—Ti.

There was a time main effect for PGEM concentration (*P* = 0.02), where values at 48 and -24 h were greater than at 8 h (Table 6). There was a drug by transport by time interaction for percent change from baseline PGEM concentration (*P* = 0.03). Sliced ANOVA revealed this interaction was due to the contrast between PLBO-CNTL steers and HEMP-CNTL steers at 48 h, with the PLBO-CNTL steers having a greater positive change in PGEM concentrations (31% versus HEMP-CNTL steers -9.3%; *P* = 0.0035).

There was a transport by time interaction for SAA (*P* *<* 0.0001), where TRANS steers at 24 and 32 h had greater SAA than TRANS steers at baseline (*P* *≤* 0.05) (Fig. 5 and Table 6).

The lower limit of quantification (LLOQ) and inter-day accuracies for each cannabinoid analyte are summarized in Table 7. Four cannabinoids—CBD-7-acid, CBDA, CBDVA, and THCA—were consistently detected across timepoints and periods (Table 8). A total of 14 cannabinoids were detected above LLOQ. Cannabidiolic acid reached the highest concentrations, followed by CBD-7-acid, THCA, and CBDVA. The modal time for observed peak concentration was 48 h for CBD-7-acid, 24 h for CBDA and CBDVA, and 32 h for THCA. One steer accounted for all positive 6-hydroxycannabidiol (CBD-6-OH), 7-hydroxycannabidiol (CBD-7-OH), THC-11-OH, and THC-acid samples. Similarly all positive CBG, CBN, 9-THC, 8-THC, and CBC samples were accounted for by a single steer; this steer and one other were the only steers with positive CBGA samples. The cannabinoids CBC, CBD-7-acid, CBDA, CBDVA, CBG, CBN, 8-THC, 9-THC, and THCA were detected in some baseline (-24 h) samples. All six HEMP steers from period 3 had detectable CBD-7-acid at baseline, and four out of six steers from period 4 had detectable CBD-7-acid at baseline, compared to only one positive steer in each of periods 1 and 2.

**Table 7.** Inter-day accuracy of QC samples and LLOQ for cannabinoids analyzed via UPLC-MS.

		Accuracy, %		
Cannabinoid	LLOQ, ng/mL	4.75 ng/mL QC	47.5 ng/mL QC	95 ng/mL QC
**8-THC**	1.0	104.3	101.3	99.4
**9-THC**	1.0	104.5	100.3	98.0
**CBC**	1.0	104.0	101.1	98.3
**CBD**	1.0	101.3	101.1	98.1
**CBD-6-OH**	2.5	86.9	94.4	101.3
**CBD-7-acid**	1.0	100.0	96.2	90.0
**CBD-7-OH**	2.5	93.7	99.4	98.8
**CBDA**	2.5	88.4	99.3	100.9
**CBG**	1.0	104.9	102.0	97.8
**CBGA**	2.5	91.2	95.6	94.6
**CBN**	1.0	104.8	102.9	101.3
**CBDV**	2.5	89.1	89.8	88.8
**CBDVA**	2.5	100.9	109.3	99.2
**THC-11-OH**	1.0	103.4	100.6	99.7
**THC-acid**	2.5	106.6	104.2	99.1
**THC-acid-glu**	1.0	100.9	102.9	102.0
**THCA**	1.0	104.2	103.2	100.5
**THCV**	1.0	91.1	96.4	95.8

**Table 8.** Cannabinoid concentrations (median; 95% CI) for holstein steers undergoing repeat transport or control events receiving IH in a 4-phase block randomized latin-square design study.^a^

	Time, h									
Cannabinoid	-24		8		24		32		48	
**Period 1**	Med	CI	Med	CI	Med	CI	Med	CI	Med	CI
**8-THC**	N/D	N/D	0	0, 8.4	0	0, 2.4	N/D	N/D	0	0, 2.4
**9-THC**	0	0, 9.1	0	0, 7.7	N/D	N/D	N/D	N/D	N/D	N/D
**CBC**	0	0, 9.8	N/D	N/D	N/D	N/D	0	0, 2.7	N/D	N/D
**CBD-6-OH**	N/D	N/D	N/D	N/D	0	0, 2.5	N/D	N/D	0	0, 2.3
**CBD-7-acid**	0	0, 1.8	1.0	0, 2.3	17.5	8.1, 28.5	20.1	10.4, 35.8	34.2	15.2, 56.5
**CBD-7-OH**	N/D	N/D	N/D	N/D	0	0, 3.3	N/D	N/D	0	0, 2.9
**CBDA**	N/D	N/D	55.8	0, 184.6	97.8	75.7, 143.8	117.5	64.9, 144.9	59.3	23.1, 103.9
**CBG**	0	0, 10.3	0	0, 9.7	0	0, 3.0	N/D	N/D	N/D	N/D
**CBGA**	N/D	N/D	0	0, 8.3	N/D	N/D	N/D	N/D	0	0, 2.3
**CBN**	0	0, 13.0	0	0, 15.4	0	0, 5.4	0	0, 2.3	0	0, 4.9
**CBDVA**	0	0, 2.3	5.4	0, 11.8	5.2	1.5, 13.2	3.6	0.3, 9.0	0	0, 3.4
**THC-11-OH**	N/D	N/D	N/D	N/D	0	0, 0.8	N/D	N/D	0	0, 1.2
**THC-acid**	N/D	N/D	N/D	N/D	0	0, 0.8	N/D	N/D	0	0, 2.1
**THCA**	0	0, 3.1	3.5	1.1, 6.3	8.1	6.4, 9.7	8.5	6.1, 11.3	5.9	1.9, 7.7
**Period 2**										
**CBD-7-acid**	0	0, 1.1	2.0	0.4, 3.5	26.9	17.9, 35.9	23.1	12.8, 38.6	42.5	34.6, 50.2
**CBDA**	0	0, 11.9	84.2	47.0, 125.4	85.0	63.1, 109.4	95.2	70.7, 150.5	40.6	32.3, 49.8
**CBDVA**	N/D	N/D	6.0	1.9, 10.0	5.0	1.4, 9.4	3.8	0.1, 7.3	0	0, 2.4
**THCA**	0	0, 4.4	3.4	0.9, 4.7	6.2	4.5, 7.6	6.7	4.6, 9.0	4.0	2.8, 5.3
**Period 3**										
**CBD-7-acid**	13.9	10.7, 18.3	13.2	9.2, 20.0	45.1	30.4, 61.9	29.4	21.7, 49.9	70.1	37.2, 96.3
**CBDA**	0	0, 5.4	59.9	41.6, 85.9	127.1	110.8, 179.4	136.2	100.0, 189.4	62.7	39.0, 93.7
**CBGA**	N/D	N/D	N/D	N/D	N/D	N/D	0	0, 1.9	N/D	N/D
**CBDVA**	N/D	N/D	5.2	1.7, 7.2	4.9	0.8, 11.3	9.5	1.0, 13.6	0	0, 3.1
**THCA**	N/D	N/D	2.9	1.8, 4.3	8.0	5.1, 10.4	8.1	5.1, 11.3	5.1	3.4, 6.8
**Period 4**										
**CBD-7-acid**	1.8	0.2, 2.6	2.2	0.7, 3.8	20.7	11.0, 32.9	22.5	14.5, 32.6	36.3	17.6, 64.8
**CBDA**	0	0, 26.9	58.4	27.3, 98.4	74.5	48.0, 142.1	69.8	30.0, 132.9	44.1	14.0, 78.9
**CBGA**	N/D	N/D	N/D	N/D	N/D	N/D	N/D	N/D	0	0, 2.5
**CBDVA**	N/D	N/D	6.1	3.1, 11.7	5.2	1.3, 11.6	5.5	1.5, 9.7	0	0, 3.8
**THCA**	0	0, 2.6	3.5	0, 14.5	6.2	4.8, 8.1	6.4	4.8, 8.0	5.7	4.1, 8.0

a An alfalfa pellet placebo or industrial hemp, dosed at 5.5 mg/kg CBDA, was given once by oral bolus immediately prior to the transport or control event (0 h). N/D: not detected.

## Discussion

This study is the first to report on the effects of concurrent IH administration and transport in cattle. A wide variety of outcome measures were evaluated to generate data for development of further studies investigating IH administration during transport. There were drug by transport interactions for the kinetic gait parameter, rear pressure, and IRT and significant drug by transport by time interactions for blood glucose and PGEM. There were transport by time interactions for a variety of outcome variables, including body weight change, accelerometry, complete blood count and serum biochemistry, cortisol, and SAA levels.

There was a significant transport by time interaction for body weight change, with TRANS steers not yet returning to their baseline (-24 or 0 h) body weight by the final collection point at 48 h. These results are consistent with previous transportation literature: cattle tend to lose 1% body weight per hour for the first three to four hours of transport and less thereafter (Coffey et al. 2001), lose the most body weight within the first 12 hours of initiation of transport (Knowles 1999; Knowles et al. 1999), and generally return to baseline body weight within 24 to 72 h following transport (Knowles et al. 1997). While the TRANS steers in the present study had not returned to baseline body weight by 48 h, there was an upward trend in body weight change (less negative change, or increased body weight) from 8 to 48 h.

The accelerometric results support prior research that has observed increased lying times in transported heifers compared to controls (Theurer et al. 2013). Conversely, studies in bulls have reported decreased lying times (Earley et al. 2013), potentially in favor of interacting with other animals (Cafazzo et al. 2012). While TRANS steers laid down more compared to baseline, similar to the findings from Theurer et al. (2013), in the present study, CNTL steers were also observed to have increased lying times. This is likely an allelomimetic response on the part of the CNTL steers, as cattle are known to synchronize lying times within a herd (Stoye et al. 2012), although this has not been specifically in non-transported cohorts. All steers in the present study had been housed together for a prolonged period prior to this study; it is possible that the stable social dynamics between the steers in the present study contributed to the changes observed in the CNTL steers following return of the TRANS cohort. While CNTL steers did follow a similar pattern to TRANS steers regarding motion index, step count, standing ratio, and lying ratio, CNTL steers had more lying bouts than TRANS steers. Thus, TRANS steers appear to stay in recumbency longer, whereas CNTL steers appear to shift between standing and recumbency. The increased lying bouts in CNTL compared to TRANS steers suggests that CNTL steers may have been more comfortable or less fatigued, and thus more willing to expend energy moving between standing and recumbent positions.

To the authors’ knowledge, there are no reports of MNT outcomes in transported cattle. The results of the present study suggest that cattle acclimate to MNT measurement over time. The observed temporal pattern of decreasing sensitivity over time is consistent with habituation to the stimulus (Grandin 1997). While sensitivity generally decreased with time (MNT values increased), left front limb MNT values for TRANS steers at 8 h were not different from baseline. One explanation for this delayed habituation in TRANS steers is the possibility of tissue damage caused by transport, which has been previously reported (Jarvis et al. 1996; Huertas et al. 2010). Tissue damage and subsequent release of prostaglandins and other inflammatory mediators may initiate a state of hyperalgesia or sensitize nerve endings in the skin or deeper tissues (Grandin et al. 2023). Further work would be needed to distinguish the temporal pattern in MNT values from the effects of transport, due to the fact that the transport period was early in the study period, when MNT outcomes were already lower than later timepoints.

This is also the first report of kinetic gait analysis being used for evaluation of transport effects. Kinetic gait analysis has previously been used to evaluate the efficacy of analgesics in cattle with induced lameness. Schulz et al. (2011) observed that cattle treated with meloxicam placed greater force on both lame and non-lame limbs compared to placebo animals. In the present study, there was a time main effect for most parameters. One explanation for the time effects is that steers were becoming habituated or more resistant to handling at 48 h than earlier in the study, resulting in shorter, slower strides with less force. Introducing cattle to the pressure mat system prior to baseline collection would allow a more direct comparison of values throughout the study. Further work is needed to determine the utility of kinetic gait analysis subsequent to transportation.

Infrared thermography has been used previously to evaluate the effects of transportation. Lei et al. (2023) investigated non-invasive markers of stress in Arouquesa cattle and found increased mean and maximum eye IRT values immediately after transport, but temperatures subsequently returned to baseline following a short rest. Brief elevations in IRT values could also be caused by elevated ambient temperatures within the transport vehicle. In the present study, left eye maximum temperatures were greatest at 8 h, which is likely due to that timepoint occurring in the afternoon. The authors believe that the drug by transport interaction observed for left eye maximum temperature percent change from baseline is largely due to the transport effect. No other IRT parameters had a significant drug main effect or interaction and the percent change from baseline was not different between HEMP-TRANS and PLBO-TRANS steers. The interaction with IH is likely due to random variation in treatment groups.

There were several changes to complete blood count and serum biochemistry parameters that are consistent with previous transportation literature, including an elevation in total white blood cell, neutrophil, and monocyte concentrations and a decrease in lymphocyte concentrations. Alterations in leukocyte numbers and function following transport result in transient changes in immune system responses (Van Engen and Coetzee 2018). Concentrations of white blood cells, neutrophils, and monocytes in all steers in the present study remained within the reference interval for our laboratory. Thus, the changes observed in the present study likely had little biological significance in regards the immunocompetence of the enrolled steers. Numerous publications have reported transport-induced neutrophilia, which is due to elevation in glucocorticoids causing decreased margination and expression of extravasation proteins (Murata et al. 1987; Earley et al. 2006; Earley and O’Riordan 2006; Buckham Sporer et al. 2008; Hulbert et al. 2011; Van Engen and Coetzee 2018). Similar to neutrophils, monocyte concentrations were also increased in TRANS steers at 8 h in the present study. Reports on monocyte concentrations following transport vary. Masmeijer et al. (2019) reported monocytosis in lightweight, but not “normal” weight, calves following transport. In a study evaluating the effects of meloxicam on stress biomarkers in transported bulls, both control and meloxicam groups exhibited elevated monocyte concentrations following transport compared to baseline (Van Engen et al. 2014). Other reports observed no change in monocyte levels (Gupta et al. 2007; Ishizaki and Kariya 2010). It is possible that age, breed, or handling differences could be responsible for the disparity in reports on monocyte concentrations. Lymphocyte concentrations were reduced in TRANS steers in the present study. Previous work has also demonstrated this reduction, which is glucocorticoid-mediated (Murata et al. 1987; Earley et al. 2006; Earley and O’Riordan 2006). As the present study was a pilot study, only immune cell concentrations were measured. Evaluation of the effects of transport on immunological function after IH administration would be an area of future interest, considering the potential anti-inflammatory effects of some cannabinoids (Pellati et al. 2018; Cosentino et al. 2023).

Not only do glucocorticoids alter immune cell concentrations, but they have also been shown to transiently increase blood glucose concentrations in transported cattle (Kenny and Tarrant 1987; Warriss et al. 1995; Browning and Leite-Browning 2013). In the present study, all steers exhibited mild hyperglycemia, with PLBO-TRANS steers at 8 h having the greatest levels. While the trends we observed do match previous transport literature [eg Kenny and Tarrant (1987)], the small numerical differences observed here are likely biologically insignificant and reflect the fact that these steers were well-accustomed to people and handling. Although there was a drug by transport by time interaction, the lack of biological significance suggests blood glucose is likely of low interest for future IH research.

While the elevations in immune cells and glucose point to transport-mediated stress, the increases observed in total protein support existing data on dehydration during transport (Jarvis et al. 1996). The transport distance used in the present study was chosen to be an intermediate transport period. Jarvis et al. (1996) found that cattle transported less than 80 mi had lower packed cell volume and total protein than those transported more than 80 miles. It has also been observed that cattle lose more body weight in high ambient transport temperatures than cooler conditions due to thermal stress (Coffey et al. 2001; Theurer et al. 2013). Total protein did not fall outside the reference interval for any steer in the present study. It is possible that, despite the prolonged transport distance and time without water, the changes in total protein were mitigated by the cooler temperatures.

The data regarding increases in white blood cells and glucose are consistent with the increased cortisol concentrations observed in TRANS steers. Cortisol exposure, as measured by AUC, was also greater for TRANS steers compared to CNTL. This stress response to transport is well-established in the literature. Buckham Sporer et al. (2008) reported a 321% increase in cortisol concentration 4.5 h into a 9 h transport period; cortisol returned to baseline by 24 and 48 h following transport. In a multiple experiment trial evaluating the effects of transport, novel environments, and loading, authors reported cortisol increased in response to transport, introduction to a novel environment, and loading (Browning and Leite-Browning 2013). That study demonstrated the multifactorial nature of transport stress, in that many aspects of typical cattle processing (loading, transport, new environment), all induced a cortisol response. The authors did not find a difference in cortisol response between cattle that were loaded and unloaded versus transported, suggesting those events are similarly stressful. In the present study, steers were returned to their home environment, so they were subjected only to the stress of loading and transport. The logarithmic mean concentrations observed in the present study study for -24, 32, and 48 h were less than 1 ng/mL, and the greatest mean concentration (averaged across treatments) was observed at 8 h (3.06 ng/mL, 95% CI: 1.83 to 5.12). Past transport studies have generally reported baseline values between roughly 2 and 15 ng/mL, with substantial increases following transport (Kenny and Tarrant 1987; Gupta et al. 2007; Buckham Sporer et al. 2008; Browning and Leite-Browning 2013). Other studies have reported reduced cortisol levels with long-distance transport and with increasing exposure to transportation (Lay et al. 1996; Van Engen et al. 2014) reported a reduction in cortisol following long-distance (16 h) transport and postulated that the decrease was due to acclimation to the stressor. While the steers in the present study were not acclimated to transport, the frequent prior exposure to handling and people could have impacted the magnitude of cortisol response. The consistently low cortisol concentrations in the present study may reflect the habituation of the steers to people and manipulation. (The authors also believe that the low cortisol concentrations observed in this study may be responsible for the large intra-assay CV, as CV increases with decreasing mean values, if SD stays the same.) Burdick et al. (2010) reported on the effects of temperament of Brahman bulls on physiologic responses to transport and found that bulls scored as ‘calm’ had lower pre- and post-transport cortisol concentrations than bulls scored as ‘temperamental’. Considering that cortisol values from the previous study by Kleinhenz et al. (2022) were also low (albeit those cattle did not undergo transport), it is possible that there is some breed or bloodline temperament effect on cortisol levels consistent across the animal sources used for that study and the present one.

In a previous study evaluating the effects of short-term IH feeding (Kleinhenz et al. 2022), hemp-fed Holstein steers had lower mean cortisol concentrations (1.59 ng/mL) than controls (5.97 ng/mL; *P* = 0.001). The hemp group had cortisol concentrations as low as 0.76 ng/mL on the last day of feeding. The authors concluded that additional work was needed, as some work has shown increased cortisol following CBD administration; however, these effects may be offset by the anxiolytic properties of some cannabinoids. There was not a significant effect of IH on cortisol in the present study; steers in the HEMP group had numerically lower AUC values than PLBO steers. Considering the previous findings from Kleinhenz et al. (2022), the handling exposure of the steers in the present study, and the small sample size of this study, the authors think it would be worthwhile to evaluate the effects of IH on cortisol with a larger cohort or in other stressful management scenarios.

To the authors’ knowledge, this is the first report of PGEM data from transported cattle. There was an overall negative change in PGEM concentration in HEMP-CNTL steers compared to a positive change in PLBO-CNTL steers at 48 h. This is consistent with a previous study, in which cattle fed IH for 14 d had an 8.8% reduction in PGEM concentrations compared to baseline versus a 10.2% increase for control cattle (Kleinhenz et al. 2022). Previous work has shown that CBD and an IH water extract have inhibitory effects on PGE_2_ production in animal and in vitro models of inflammation (Di Giacomo et al. 2021; Costa et al. 2004). Furthermore, cannabinoids, including CBD, CBDA, THCA, CBG, CBGA, and IH leaf and inflorescence extracts have been shown to decrease cyclooxygenase production in *ex vivo* and in vitro inflammatory models (Ruhaak et al. 2011; Costa et al. 2004; Shin et al. 2024). Specifically, THCA, CBG, CBGA have been shown to have COX-2 inhibitory properties (Ruhaak et al. 2011); results for CBDA have been mixed, with some studies showing inhibition (Takeda et al. 2008) and others showing an increase in COX-2 (Ruhaak et al. 2011). Bartkowiak-Wieczorek et al. (2025) showed dose-dependent increases in COX-1 expression in rat brain following administration of two IH extracts and variable changes in COX-2 expression based on brain region. The effect observed in this study, complicated by the three-way interaction, suggests that further work is needed to confirm the effects of PGEM in cattle.

Two acute phase proteins (APP), SAA and fibrinogen, were evaluated in the present study. In a study evaluating the effects of transport and commingling on APP in newly weaned calves, Arthington et al. (2003) found a tendency for SAA to be increased in transported calves (48.9 vs. 33.4 µg/mL in nontransported calves). However, these results are not straightforward to interpret, as there was not an unweaned control group. Thus, the relative contribution of transport versus weaning on the APP response in these calves is unknown. In a study by Lomborg et al. (2008), Holstein cows and heifers were subjected to a combination of transport, social isolation, and an uncomfortable environment. The authors reported significantly greater SAA in all animals at 48 h compared to 0 h, with a range of 30 to 482 µg/mL SAA at 48 h. While there was a transport by time interaction in the present study, all SAA values were much lower, with the greatest average concentration in TRANS steers at 24 h (20.8 µg/mL, 95% CI: 8.3 to 52.3). As discussed previously, these lower values could be due to the level of prior human socialization of the steers in the present study. Another possible explanation is that there are differences in baseline APP profiles or the APP response to stress due to age, sex, or breed. There was no effect of IH on SAA levels in the present study, consistent with a previous investigation of stress biomarkers after a 14-d period of feeding IH (Kleinhenz et al. 2022). However, in both the present study and the study by Kleinhenz (2022), animals were free of disease or other diagnosed inflammatory conditions. It is unknown if IH may impact SAA in light of a true inflammatory impetus. A study by Winders et al. (2024) found that cattle fed hempseed cake had lower interleukin 1-α, tumor necrosis factor-α, and interleukin 36 receptor antagonist after LPS challenge than cattle receiving a control diet or a diet with dried distillers grains plus solubles. This suggests that hempseed cake may have anti-inflammatory properties. Further work is needed to evaluate the anti-inflammatory potential of different hemp products.

In the present study, 4 cannabinoids, CBD-7-acid, CBDA, CBDVA, and THCA, were consistently detected above the LLOQ. In a previous study investigating the pharmacokinetics of cannabinoids in cattle following a single oral dose of IH, CBDA, THCA, CBDVA, and CBCA were detected in all samples and CBD in only a few samples (Kleinhenz et al. 2020a). In another study investigating a 14-d IH feeding period on plasma cannabinoid concentrations and effects on behavior and stress in cattle, CBDA, CBD, CBDVA, CBCA, CBGA, and THCA were detected (Kleinhenz et al. 2022). Smith et al. (2023) detected sporadic and low levels of CBDA/THCA, CBDVA, and CBNA in plasma samples from heifers fed hempseed cake for 111 d; the quantification methods used in that study were unable to differentiate between CBDA and THCA. In a recent study evaluating the depletion of cannabinoids in cattle fed IH leaves, Fruge et al. (2025) detected CBD-7-acid, CBD, CBDA, CBDVA, THCA, and THCVA. Irawan et al. (2025) detected CBC, CBCA, CBD, CBDA, CBG, 9-THC, THCA, and THC-11-OH in plasma of cows fed spent hemp biomass.

The modal time of peak CBDA concentrations (24 h) in the present study is later than the time to maximum CBDA concentration previously reported [11.8 h (Kleinhenz et al. 2020a) and 16.7 h (Kleinhenz et al. 2022)]. Fruge et al. (2025) reported maximum observed concentrations of CBD-7-acid at 72 h, as compared to 48 h in the present study. That study involved 14 d of IH leaf administration, but plasma was only collected for the first 72 h. Because the present study was not designed to determine pharmacokinetic parameters and samples were only collected through 48 h, the observed time of maximum cannabinoid concentrations may not be the time of the true peak concentrations.

The varying cannabinoid signatures and pharmacokinetic profiles in the present study compared to previous work could be due to cultivar differences across studies. However, it is also possible that altered rumen pH or other physiological parameters could play a role in changing cannabinoid absorption. Fruge et al. (2025) reported large variability in fecal cannabinoid profiles in cattle fed IH leaves, postulating that there were marked differences in individual animals’ capacity to absorb or metabolize cannabinoids. The different cannabinoid signatures across studies could be due to individual animal differences. In addition, individual variability in metabolic or absorptive capacity could explain why some cannabinoids (including 9-THC) were only detected in one or two steers in the present study. Further investigation is needed to establish pharmacokinetic data for individual cannabinoids and IH forms and to illuminate the cause of the reported variation in individual animals’ cannabinoid profiles.

The cannabinoid panel used in the present study evaluated CBD-7-acid concentrations. This cannabinoid has only been reported in one other study in cattle, which evaluated cannabinoid depletion in cattle fed IH leaves (Fruge et al. 2025). Although considered an inactive metabolite in people, CBD-7-acid is the major metabolite of CBD and has a long half-life, ranging from 22 to 33 h in humans (FDA CDER 2017; Taylor et al. 2018). In a study investigating the pharmacokinetics of CBD and its metabolites following CBD paste administration in horses, the authors reported the terminal half-life for CBD-7-acid was 79.85 h (Eichler et al. 2023). Fruge et al. (2025) observed the greatest concentrations of CBD-7-acid at 72 h after the first of 14 daily doses; however, they did not sample past 72 h. The FDA has stated an interest in CBD-7-acid, as it is considered a disproportionate metabolite in people (FDA CDER 2023). In the present study, samples were only collected intermittently up to 48 h after IH dosing, so an estimate of time to peak concentration or elimination half-life is impossible. There were detectable levels of CBD-7-acid in baseline samples from all six steers in period 3 and four of six steers in period 4. Steers receiving IH in period 3 had previously received IH in period 2, and steers receiving IH in period 4 had previously received IH in period 1. The baseline CBD-7-acid concentrations from period 3 were greater than period 4, but this difference was not statistically analyzed. All steers underwent at least a 10-d washout period between periods, suggesting that CBD-7-acid has a very prolonged half-life in cattle. Combined with data from Fruge et al. (2025), it appears that the elimination half-life of CBD-7-acid in cattle is long, as in people and horses.

In addition to CBD-7-acid, other cannabinoids were sporadically detected in baseline samples. These other cannabinoids did not follow the temporal trend of the CBD-7-acid baseline samples. For CBC, CBG, CBG, 8-THC, and 9-THC, all positive samples came from one steer from period 1. The samples from this steer were analyzed at the beginning of a UPLC-MS/MS run following analysis of the calibration curve and a methanol wash. While it is possible that these large, neutral cannabinoids were not sufficiently removed from the column following the wash, resulting in false positive results, there were no cannabinoids detected in the methanol wash. The authors feel it is more likely that there were some early-eluting compounds or matrix interference producing these results. However, as previously mentioned, it is possible that individual animal variation in cannabinoid metabolism could explain the positive results from this one steer.

Limitations of this study include the small sample size and the steers having extensive previous handling and human interaction. This prior exposure to people and manipulation during trials may have reduced the steers’ stress response. Another limitation of this study is that the dosing regimen may not have allowed time for cannabinoids to reach effective concentrations during the transport period. In the previous report on the plasma pharmacokinetics of CBDA, time to maximum concentration was 11.8 h (Kleinhenz et al. 2020a). Pharmacodynamic studies evaluating effective cannabinoid concentrations or times to reach effectiveness for specific outcomes in cattle have not been reported. While the dosing regimen chosen may have precluded some pharmacodynamic changes from occurring during the transport period itself, there were complex effects of IH on blood glucose and prostaglandin concentrations. Interaction with transport and time effects makes interpretation of these results difficult. While the single dose can be viewed as a limitation, this regimen was chosen by the authors because it represents the most practical situation for producers, should further research (and approval of IH products for cattle) indicate that IH may have use as a therapeutic agent for alleviating negative effects of transport. Performing a study to evaluate these same outcomes under steady-state conditions would be enlightening, as one of the main goals of IH research in livestock is to pursue IH products as feed ingredients. Animals eating a diet containing IH would presumably be exposed for extended periods and would reach steady-state concentrations of cannabinoids.

As the IH market continues to grow, investigating potential therapeutic applications of IH and IH byproducts in cattle production is of interest to stakeholders. This study evaluated a wide range of outcome variables: body weight, accelerometry, kinetic gait analysis, mechanical nociceptive threshold (MNT), infrared thermography (IRT), complete blood count (CBC), serum biochemistry, blood cortisol, prostaglandin E_2_ metabolite (PGEM), and serum amyloid A (SAA). In conjunction with transport and time, IH resulted in reductions in PGEM compared to placebo treatment. Transport resulted in predictable decreases in bodyweight and lymphocyte concentrations and increases in neutrophil, blood glucose, total protein, cortisol, and SAA concentrations. Transported steers had altered activity patterns and transiently increased tissue sensitivity as assessed via MNT when compared to non-transported steers. The results from this study can serve as a basis for the development of future studies regarding transportation and associated stress in cattle.

## Abbreviations

8-THC: Δ8-tetrahydrocannabinol
9-THC: Δ9-tetrahydrocannabinol
AUC: area under the curve
CBC: cannabichromene
CBCA: cannabichromenic acid
CBD: cannabidiol (CBD)
CBD-6-OH: 6-hydroxycannabidiol
CBD-7-acid: (−)-7-nor-7-carboxy cannabidiol
CBD-7-OH: 7-hydroxycannabidiol
CBDA: cannabidiolic acid
CBG: cannabigerol
CBGA: cannabigerolic acid
CBN: cannabinol
CBDV: cannabidivarin
CBDVA: cannabidivarinic acid
IH: industrial hemp
IRT: infrared thermography
LLOQ: lower limit of quantification
MNT: mechanical nociceptive threshold
PGEM: prostaglandin E2 metabolite
QC: quality control
SAA: serum amyloid A
THC-11-OH: ( ±)-11-hydroxy-Δ9-tetrahydrocannabinol
THC-acid: (−)-11-nor-9-carboxy-Δ9-tetrahydrocannabinol
THC-acid-glu: (+)-11-nor-9-carboxy-Δ9-tetrahydrocannabinol glucuronide
THCA: Δ9-tetrahydrocannabinolic acid A
THCV: Δ9-tetrahydrocannabivarin
UPLC-MS/MS: ultra-performance liquid chromatography triple quadrupole mass spectrometry
